# PRP pre-treatment of the implantation zone improves the survival rate of fat autograft

**DOI:** 10.3389/fbioe.2025.1545419

**Published:** 2025-04-11

**Authors:** Ilona Pak, Meirambek Askarov, Dmitriy Klyuyev, Min Sungh Tak, Ulpan Batenova, Dauren Yeskermessov, Yevgeniy Kamyshanskiy

**Affiliations:** ^1^ Plastic Surgery Department, Karaganda State Medical, Karaganda, Kazakhstan; ^2^ Life Science Institute, Karaganda State Medical, Karaganda, Kazakhstan; ^3^ Plastic and Reconstructive Surgery Department, Soonchunhyang University Hospital, Seoul, Republic of Korea; ^4^ Pathology Department, Karaganda State Medical, Karaganda, Kazakhstan

**Keywords:** preimplantation preparation, Lipofilling, fat autograft, platelet-rich plasma, morphological characteristic

## Abstract

**Background:**

Lipofilling is gaining in popularity daily as a method of replenishing the volume of almost any part of the human body. However, the use of adipose tissue as a filler has its limitations in the long term, in the form of a low survival rate of the fat graft because of fibrotic replacement and fat cell apoptosis. The aim of this study was a comparative morphological assessment of fat autograft survival in the groups undergoing a standard lipofilling protocol and the pre-implantation treatment of the implantation area with platelet-rich plasma.

**Material and methods:**

Twenty-four male Californian rabbits that had undergone hypodermic implantation of a fat autograft in the area of the auricle were used in the study. All cases were classified into three groups depending on the method of platelet-rich plasma treatment. After 3 months (90 days) of exposure, macroscopic and histological examinations of the fat autograft were conducted.

**Results:**

The volume and histological normality of the fat autograft were statistically significantly preserved in the group with preoperative treatment of the implant area and intraoperative treatment of the autograft compared to the group without it and with intraoperative treatment alone.

**Discussion:**

We have demonstrated that the pre-implantation use of platelet-rich plasma significantly improves the standard intraoperative technique and increases the survival rate of the fat autograft by enhancing angiogenesis, with a decreased degree of hypoxic-ischemic, fibrotic, and inflammatory damage in the implant area.

**Conclusion:**

The effect of improved preservation of the morphological pattern of the fat implant during preoperative treatment may be due to a favorable preoperative locoregional stromal-vascular microenvironment with an active perfusion and diffusion potential of the stromal skeleton.

## 1 Introduction

The primary drawback of fat allotransplantation is the unpredictable resorption of the graft, which in some cases necessitates repeated surgeries. Three months post-operation, the volume of the residual fat graft varies from 20% to 80%–90% of the initially injected volume (Doornaert et al., 2018; Shih et al., 2020).

Several hypotheses have been proposed to explain the mechanisms of adipocyte necrosis and resorption in transplanted fat. These include the graft survival theory (Peer, 1950), the graft replacement theory (Rasmussen et al., 2017; Allen et al., 2013), and the host cell replacement theory (Oranges et al., 2019; Doi et al., 2015). Each of these theories highlights different factors influencing graft stability while also emphasizing the importance of preparing the recipient site.

Methods for preparing the implantation site include physical and mechanical stimulation of tissues, which creates favorable conditions for angiogenesis induction (Kim et al., 2023; Giatsidis et al., 2017; Wei et al., 2019; Zhang et al., 2024; Lujan-Hernandez et al., 2016), implantation of alloplastic materials (Baran et al., 2002), preconditioning of the recipient site with biologically active substances (Koh et al., 2011; Topcu et al., 2012; Yildiran et al., 2022), ischemic preconditioning (Gassman et al., 2016; Cha et al., 2022), and microneedling (Sezgin et al., 2014; Righesso et al., 2021). Among these various approaches, the combined use of platelet-rich plasma (PRP) is considered a promising and practical method (Jin et al., 2013; Modarressi, 2013). PRP is a concentrated plasma derived from the patient’s blood, containing higher concentrations of platelets than whole blood. Current evidence-based applications of PRP include the treatment of chronic ulcers (Salgado-Pacheco et al., 2024), use in maxillofacial surgery (Anitua et al., 2021; Arthur Vithran et al., 2023), orthopedic and trauma surgery (Zhu et al., 2024; Gharpinde et al., 2024; Koyanagi et al., 2023), and cosmetic and plastic surgery (Rahman et al., 2024; Li et al., 2025; White et al., 2021). In addition to promoting neovascularization, the growth factors in PRP exert cytoprotective and regenerative effects on the fat graft and its cellular components. PRP is often described as a “natural reservoir” of regenerative factors that can enhance tissue repair (Alves and Grimalt, 2018; Vladulescu et al., 2023).

We hypothesized that the administration of PRP into the recipient site 24 h before implantation could accelerate angiogenesis and activate revascularization mechanisms, thereby creating better conditions for graft survival during the ischemic phase post-transplantation. Similar to how an athlete warms up muscles before exercise to improve functionality and prevent injury, PRP could serve as a “warm-up” for the recipient tissues. Its preliminary introduction activates regenerative processes, improves blood supply, stimulates capillary growth, and reduces the risk of ischemic cell damage, potentially enhancing graft stability and survival.

The aim of this study is to investigate the effects of preoperative PRP treatment at the implantation site and intraoperative addition of PRP to the fat graft on graft survival, as well as its macroscopic and histological characteristics, in experiments conducted on rabbits.

## 2 Materials and methods

### 2.1 PRP preparation

**FIGURE 1:**
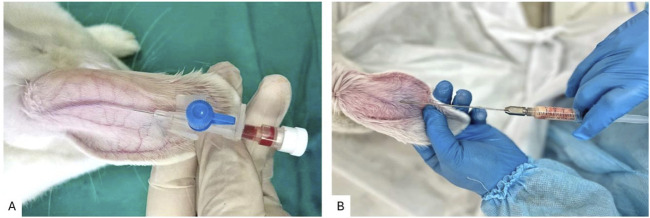
**(A)** Technique for collecting venous blood from the central auricular vein of a rabbit; **(B)** Technique for injecting autologous fat graft into the rabbit ear.

PRP was obtained using standard laboratory test tubes with a volume of 10 mL, to which 1 mL of 3.8% sodium citrate was added as an anticoagulant. 9 mL of peripheral blood was collected from the venous vessel of the rabbit ear (Figure 1A) and centrifuged for 10 min at 1700 g. The middle layer, containing platelet-rich plasma (PRP), was isolated and subsequently centrifuged at 800 *g* for an additional 10 min to separate the PRP pellet from platelet-poor plasma. This procedure yielded PRP with a platelet concentration of 1.2 × 10^8^ cells/mL (Hersant et al., 2018).

### 2.2 Animals and surgical procedures

The study used 24 male mongrel rabbits aged 5–6 months with a body weight of 3.1–3.5 kg. All procedures involving animals were conducted in accordance with the guidelines of DIRECTIVE 2010/63/EU on the protection of animals used for scientific purposes (European Parliament and Council, 2010) and were approved by the Animal Care Committee of the University.

The animals were housed in a vivarium under standard controlled conditions. They were individually placed in polypropylene cages to maintain a temperature of 22°C ± 2°C, a 12-h light/dark cycle, and a minimum relative humidity of 40%. The rabbits were fed *ad libitum*, with free access to water. In adherence to the bioethical principles of Replacement, Reduction, and Refinement (the 3Rs) as outlined by Russell and Burch, the sample size for animal experiments in this study was determined as the minimum number of animals required to obtain statistically significant results (Cruz-Orive and Weibel, 1990).

**FIGURE 2:**
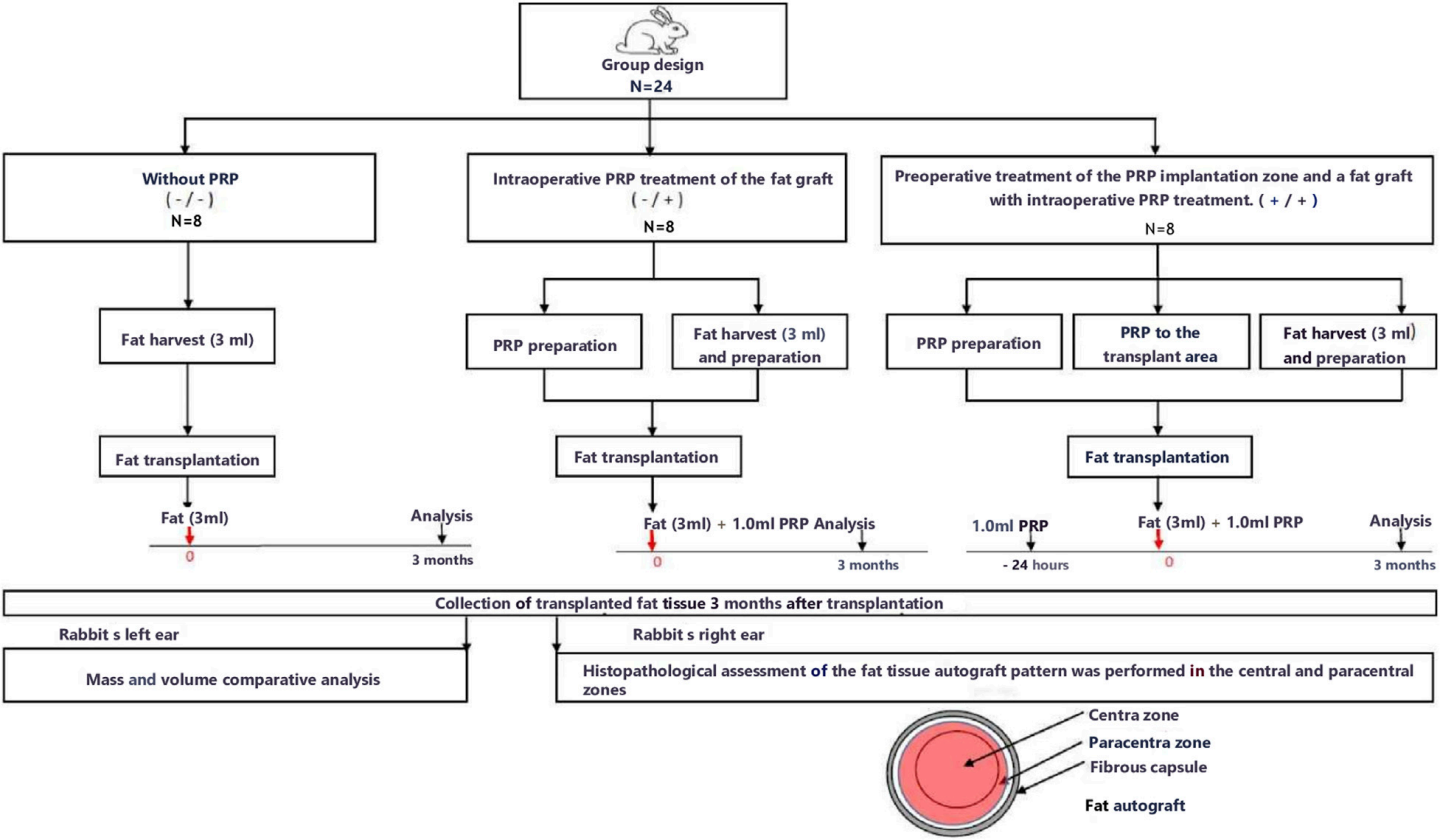
Study design.

A research flow diagram is shown in Figure 2.

The animals were randomly divided into three equal groups of eight animals.

- Group without PRP (Group 1) – the fat graft and the implantation site without PRP treatment;
- The PRP group (Group 2) – only intraoperative PRP treatment of the fat graft and without preoperative PRP treatment of the implantation site;
- PRP group (Group 3) - preoperative treatment of the PRP implantation zone (24 h before the operation, 1 mL of PRP was injected into the implantation zone) and a fat graft with intraoperative PRP treatment.

To obtain autologous fat, the animal was placed in a supine position with its limbs secured, and the hair was removed from the inguinal regions. Following premedication and sedation, the surgical site was prepared with antiseptic treatment, and up to 5 mL of Klein’s solution was injected into the fat harvesting area on each side. The solution was allowed to remain in place for 15 min. Subsequently, in the inguinal region, corresponding to the location of the fat pads, punctures were made in the soft tissues using an N11 scalpel. Coleman cannulas (Mentor, United States) were utilized: a universal blunt-tipped cannula for lipoaspiration with one central and two lateral apertures (3 mm in diameter, 15 cm in length) and an N1 infiltration cannula (blunt-tipped) (1 mm in diameter, 9 cm in length).

Fat tissue harvesting was performed using the Coleman technique with the universal lipoaspiration cannula and 10 mL syringes *via* manual aspiration. The syringes containing the lipoaspirate were sealed with Luer-Lok caps (Tulip, United States), after which the plungers were removed. The syringes were centrifuged for 3 min at 3,000 rpm (800 g). Following centrifugation, the lower layer was drained, leaving the cellular fraction. After processing the autograft, the fat tissue was weighed using a laboratory scale (Shimadzu AY120), with the average mass of the fat tissue reaching 3 g. The collected fat tissue was then transferred into 1 mL syringes. The incision site was closed with a single interrupted suture (Vicryl 5/0).

Rabbit ears were selected as a recipient area for the implantation of a fat autograft in the area between cartilage and skin. The technique for autologous fat grafting into the rabbit ear is illustrated in Figure 1B. The minimal subcutaneous tissue in the ear creates a model that closely approximates real clinical conditions, while also allowing for standardization of the injection depth of the graft, eliminating anatomical variations inherent to other regions. The tight adherence of the skin to the cartilage minimizes graft displacement. This enables precise comparisons between study groups, ensuring standardized experimental conditions, reducing variability due to recipient tissue heterogeneity, and facilitating graft excision. The ischemic microenvironment replicates the initial ischemic phase following fat grafting, which is particularly relevant to our study as it allows for an objective assessment of the impact of preoperative PRP preparation of the recipient site on angiogenesis, reduction of fibrotic changes, and enhancement of graft survival.

In Group 1 (fat graft and implantation site without PRP treatment), after antiseptic preparation of the planned implantation site, punctures were made in the soft tissues at the center of the left and right ears using an N11 scalpel. Purified fat tissue (3 g) was transplanted into the subcutaneous layers of both ears using a 1 mL syringe and an N1 infiltration cannula in a linear technique. The surgical wounds were closed with a single interrupted suture (Vicryl 5/0).

In Group 2 (intraoperative PRP treatment of the fat graft without preoperative PRP treatment of the implantation site), after antiseptic preparation of the planned implantation site, punctures were made in the soft tissues at the center of the left and right ears using an N11 scalpel. To prepare the PRP, 9 mL of venous blood was collected from the ear vein into a tube containing 1 mL of 3.8% sodium citrate. The purified fat tissue (3 g) was mixed with 1 mL of PRP obtained after a two-step centrifugation process. The mixture of fat autograft and platelet-rich plasma was transplanted into the subcutaneous layers of both ears using a 5 mL syringe and an N1 infiltration cannula in a linear technique. The surgical wounds were closed with a single interrupted suture (Vicryl 5/0).

In Group 3 (preoperative PRP treatment of the implantation site and PRP treatment of the fat graft), to prepare the PRP, 9 mL of venous blood was collected from the ear vein into a tube containing 1 mL of 3.8% sodium citrate. Preoperative PRP treatment of the implantation site was performed 24 h before fat grafting. A total of 1 mL of PRP, obtained after a two-step centrifugation process, was injected subcutaneously into the planned implantation site using a papular technique with 0.5 cm intervals. Twenty-four hours after PRP injection, following antiseptic preparation of the implantation site, punctures were made in the soft tissues at the center of the left and right ears using an N11 scalpel. The purified fat tissue (3 g) was mixed with 1 mL of PRP obtained after a two-step centrifugation process. The mixture of fat autograft and platelet-rich plasma was transplanted into the subcutaneous layers of both ears using a 5 mL syringe and an N1 infiltration cannula in a linear technique. The surgical wounds were closed with a single interrupted suture (Vicryl 5/0).

Outcome evaluation was conducted 12 weeks after the surgical procedure. The 3-month interval was chosen as the endpoint because studies have identified this period as representative for evaluating the long-term stability of the graft following the completion of the “acute” regenerative and adipogenic phases of fat graft survival (Bourne et al., 2021; Loder et al., 2024).

### 2.3 Macroscopic examination

Fat autografts were examined and photographed in the operating room immediately after excision. Each animal’s left ear fat autograft was separated from fibrous tissue and fascia. After visual macroscopic assessment, the volume of the fat graft was measured by immersing it in a measuring tube with saline (Thanik et al., 2009; Torio-Padron et al., 2011; Minn et al., 2010). Then, this volume was weighed using a benchtop electronic balance.

### 2.4 Histological examination

#### 2.4.1 Preparation and processing of research objects

The object of histological examination is a fatty graft excised from each animal’s contralateral (right) ear. The tissues were fixed in neutral buffered 10% formalin (Biovitrum, Italy) at 4°C for 24 h. The tissue samples were labeled in such a way that the researchers were aware only of the animal number and the postoperative day, but not the study group. After fixation, the tissue samples were rinsed with distilled water and dehydrated through a series of increasing alcohol concentrations, followed by immersion in xylene and embedding in paraffin blocks. Tissue sections of 3–4 µm thickness were obtained using a microtome and mounted on glass slides. The slides were then deparaffinized and stained with hematoxylin and eosin (H&E) and Masson’s trichrome. A mounting medium was applied to each section, covered with a coverslip, and allowed to dry. The stained sections were examined under a light microscope.

For hematoxylin and eosin staining, the tissue sections were immersed in Mayer’s hematoxylin for 15 min and then rinsed with water for 5 min. Subsequently, the sections were stained with eosin for 1 min. For Masson’s trichrome staining, a commercially available kit (Trichrome Stain (Masson) Biovitrim TU 9398-001-89079081-2012) was used according to the standard protocol. Masson’s trichrome was employed to identify fibrous tissue and blood vessels.

#### 2.4.2 Histomorphometric assessment of histological pattern, inflammation, and angiogenesis

Morphometric analysis was conducted by two independent researchers experienced in working with animal models, who were blinded to the group allocation and the specific interventions performed.

Histomorphological analysis was carried out using a Zeiss AxioLab 4.0 microscope equipped with a digital color microphotography system and ImageJ software for morphometric evaluation.

Hematoxylin and eosin staining were used to determine the general morphological pattern to assess inflammation and angiogenesis. For the histological evaluation of fibrosis, Masson trichrome staining was used, which detects an increased amount of collagen.

Histopathological analysis was performed on a longitudinal section of fat tissue, including a central “necrotic” zone and a paracentral “regenerating” zone for each sample in each group at ×400 magnification.

For each tissue section, three patterns were analyzed: “*physiological adipose tissue*” – adipose tissue without morphological abnormalities, consisting of adipocytes of regular shape and uniform size; “*cystic degeneration*” – adipose tissue composed of small/medium-sized cysts and adipocytes with larger diameter compared to normal adipocytes and irregular shape; and “*fibrous tissue*” – amorphous and disorganized connective tissue. This assessment was expressed as a percentage of the total area of the central and paracentral zones of the autologous fat graft.

Polymorphonuclear neutrophils, lymphocytes, and macrophages were counted in five fields of view at ×200 magnification.

Angiogenesis was assessed by calculating the average number of vessels for each sample in each group’s central and paracentral area by 10 visual fields at magnification ×200.

### 2.5 Statistical analysis

Statistical analysis was performed using the χ2 test corrected for continuity (Y-test) using the Mann-Whitney test. Statistical data processing was performed using IBM SPSS 22.0 (IBM Corp., Armonk, N.Y.). The differences were considered statistically significant at p ≤ 0.05.

## 3 Results

### 3.1 Macroscopic study of fat autograft survival

**FIGURE 3:**
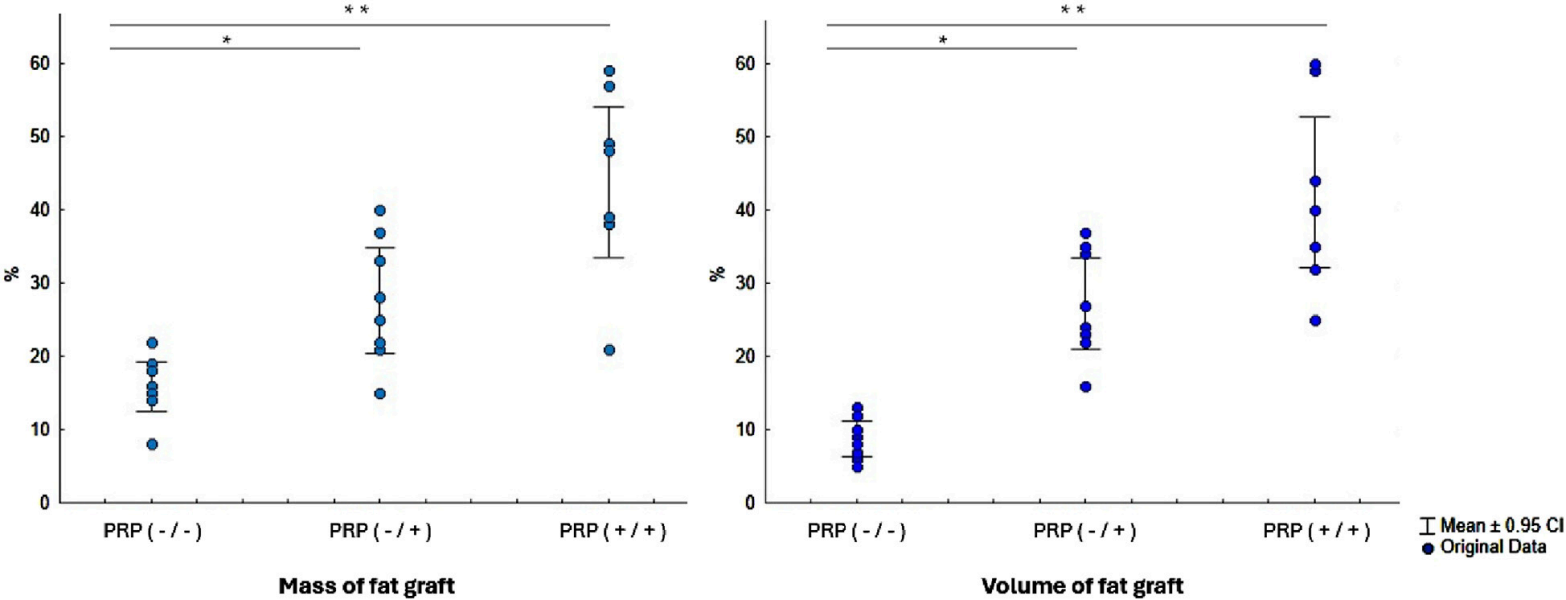
Comparative analysis of fat graft mass and volume three months after implantation with and without PRP treatment of the fat graft and its implantation site. Abbreviations: PRP (-/-) - fat graft and implantation site without PRP treatment, PRP (-/+) - only intraoperative PRP treatment of the fat graft without preoperative PRP treatment of the implantation site, PRP (+/+) - preoperative PRP treatment of the implantation site and PRP treatment of the fat graft. *- Statistically significant difference PRP (-/+) versus PRP (-/-) group (p < 0.01); **- Statistically significant difference PRP (+/+) versus PRP (-/-) and PRP (-/+) groups (p < 0.01).

Macroscopic examination of the fat autografts 3 months after transplantation revealed significant morphological changes in the study groups (Figure 3). All animals underwent the surgical procedure without postoperative complications. Throughout the observation period, there were no signs of acute inflammation, abscess formation, serous fluid accumulation, or tissue necrosis in either the implant area or the surrounding tissues. However, the degree of preservation of graft mass and volume varied significantly depending on the presence and method of PRP treatment.

In the group without PRP, the grafts exhibited pronounced signs of resorption, loss of volume and mass, dense consistency, and indistinct borders. The graft tissue was heterogeneous, combining areas of yellowish-white tissue, pink and red hues, with focal gray fibrous strands. According to electronic scales, the grafts in this group lost more than 85% of their initial mass, retaining only 1/7 of the original mass (15.5 [14.75–18.25] %). The reduction in volume was even more pronounced, exceeding 90% (retention of 1/12, 8.5 [6.5–10.5] %).

In the group with intraoperative PRP treatment of the autograft, the morphological appearance of the grafts was characterized by a more homogeneous structure and well-defined borders. The graft tissue was predominantly white-yellow in color, with areas of whitish inclusions and a finely nodular surface. The consistency of the grafts was moderately dense, with reduced elasticity compared to intact adipose tissue. The macroscopic appearance of the fat autograft showed preservation of approximately 1/4 of its mass and volume (26.5 [21.75–34.0] % and 25.5 [22.8–34.3] %, respectively).

In the group with combined PRP preparation, which included both preoperative treatment of the implantation site and intraoperative treatment of the graft, the macroscopic appearance was the most favorable. The grafts maintained clear borders, a homogeneous structure, and elasticity. The color of the grafts was uniform, yellow-white, without areas of significant fibrosis or necrosis. All grafts retained approximately half of their initial mass and volume (43.5 [38.75–51.0] % and 42.0 [34.25–47.75] %, respectively).

### 3.2 Histological examination

#### 3.2.1 Comparative histomorphometric assessment of the histological pattern of a fat autograft in experimental study groups

**FIGURE 4:**
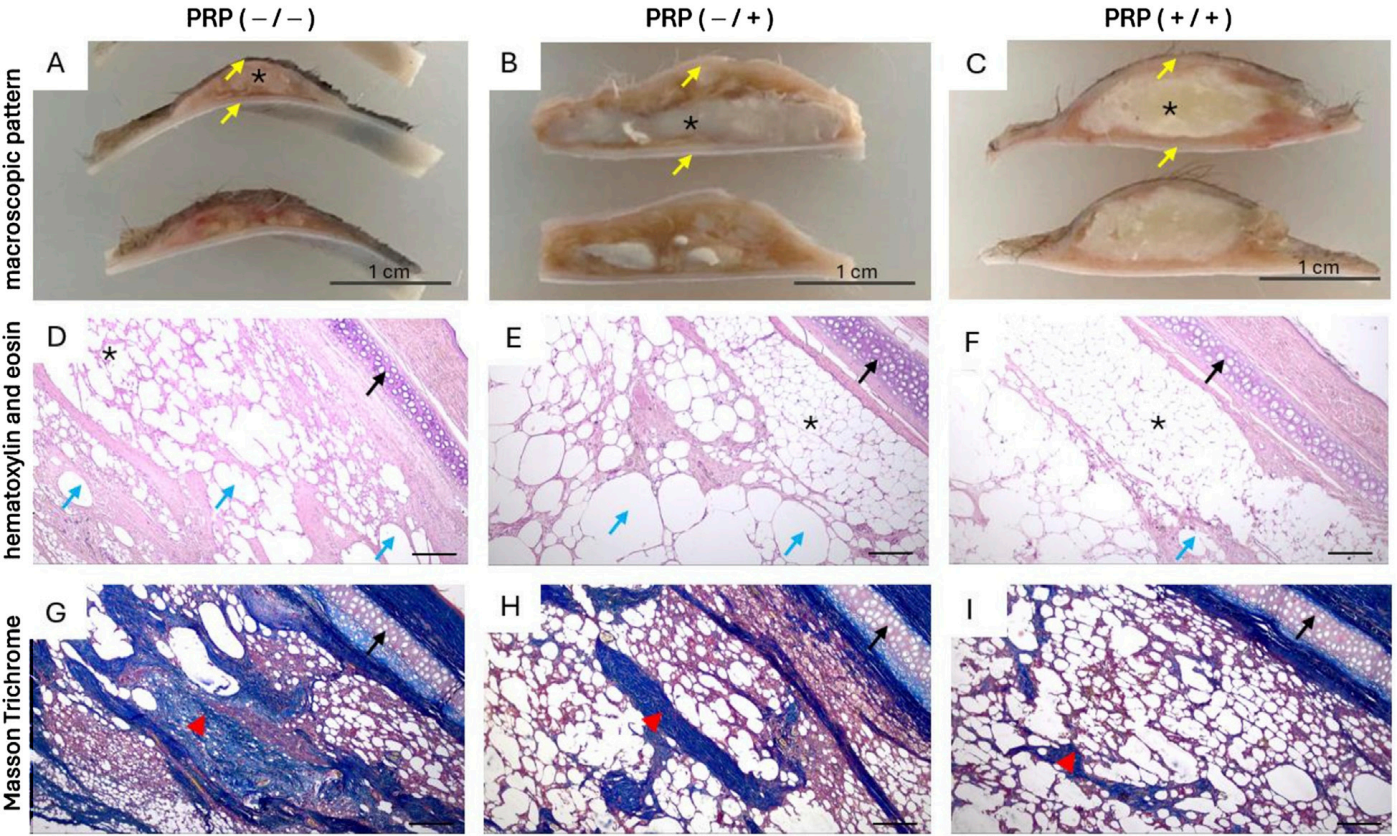
Macroscopic and histological patterns of autologous fat grafts with and without PRP treatment three months after lipotransplantation. Abbreviations: * - fat graft, yellow arrow - skin, black arrow - cartilage, blue arrow large fatty vacuoles and vacuolated cysts formed from destroyed adipocytes, red arrowhead - fibrous tissue. **(A, D, G)** - Without PRP: **(A)** Pronounced graft resorption, with the preserved fragment represented by an irregularly shaped bolus lacking clear borders, grayish-white in color with areas of pinkish-red hue. The cut surface is heterogeneous, finely nodular, with focal depressions and proliferation of thread-like fibers of grayish-white color. **(D, G)** Diffuse fibrosis, characterized by amorphous and disorganized connective tissue with scattered adipocytes and large fatty vacuoles formed from destroyed adipocytes, along with focal accumulations of immune inflammatory cells. Scale bar 500. **(B, E, H)** Only intraoperative PRP treatment of the fat graft: **(B)** flattened fat bolus, yellowish-white in color, with areas of whitish inclusions and a finely nodular surface. **(E, H)** Physiological fat tissue with focal cystic degeneration and cord-like growths of connective tissue exhibiting myxoid degeneration and bundles of thin-walled microvessels. Scale bar 500. **(C, F, I)** Preoperative PRP treatment of the implantation site and PRP treatment of the fat graft: **(C)** An oval- shaped graft, yellowish-white in color, with uniform staining, homogeneous structure, and clear borders. **(F, I)** Predominantly physiological fat tissue without morphological abnormalities, consisting of adipocytes with uniform shape and size, with single cysts, microvessels, and focal growths of connective tissue. Scale bar 500.

In the group without PRP, the histological picture in the central and paracentral zones of the fat autograft was characterized by an absolute predominance of fibrous tissue over adipose tissue (Figure 4). The mean area of fibrous tissue was 38.5% (IQR: 34.5%–42%), indicating large-focal and diffuse lesions. Physiological adipose tissue was preserved focally, with a mean area of 21.5% (IQR: 18%–30%). In most fields of view across all histological sections, the number of intact adipocytes was minimal. The preserved adipocytes had a typical structure—a single large lipid droplet occupying almost the entire cell volume and displacing the nucleus to the periphery. Significant cystic degeneration was observed, with numerous small and medium-sized cysts localized in areas where adipose tissue was replaced by connective tissue. The mean area of cystic degeneration was 40% (IQR: 31.8%–41.3%). The foci of cystic degeneration were formed from destroyed adipocytes and alternated with dense connective tissue structures.

In the group with only intraoperative PRP treatment, the histological picture in the central and paracentral zones of the fat autograft was characterized by a predominance of adipose tissue over fibrous tissue. The adipocytes retained their regular round shape and uniform size. The mean area of physiological adipose tissue was 47.5% (IQR: 37.3%–58%): in one case (12.5%), physiological adipose tissue was the predominant component; in five cases (62.5%), a diffuse pattern of adipose tissue was observed; and in two cases (25%), focal distribution of adipose inclusions was noted. Cystic degeneration was widespread, with a mean affected area of 32.5% (IQR: 27.3%–36.3%) and the formation of small and medium-sized cysts in the central and paracentral zones of the graft. Fibrous tissue was represented by isolated connective tissue strands with signs of myxoid degeneration, predominantly located in the central zone of the graft. The mean area of fibrous tissue involvement was 15.5% (IQR: 12.5%–25.3%), consistent with minimal and focal damage. Minimal damage was observed in six cases (75%), and focal damage in two cases (25%).

In the group with PRP treatment of both the graft and the implantation site, the histological picture in the central and paracentral zones of the fat autograft was characterized by a high degree of preservation of physiological adipose tissue with minimal signs of remodeling. The mean area of physiological adipose tissue was 79% (IQR: 69.5%–86.5%). Cystic degeneration was moderate, with a mean affected area of 16% (IQR: 9.8%–22.8%). Fibrotic changes were minimal. The mean area of fibrotic tissue involvement was 4.5% (IQR: 2.8%–10%), consistent with absent or minimal damage. In four cases (50%), fibrosis was almost undetectable in the paracentral zone of the fat autograft (less than 5%). In the remaining cases, small focal proliferations of connective tissue were observed.

#### 3.2.2 Comparative histomorphometric characteristics of the reactive inflammatory pattern

Histological analysis did not reveal polymorphonuclear granulocytes in any of the study groups.

In the group without PRP, scattered lymphocytic and macrophage infiltration was observed, represented by immune cells diffusely distributed throughout the thickness of the graft, with a median count of 8.5 cells (IQR: 6.75–23.3). In the group with intraoperative PRP treatment, lymphocytic and macrophage infiltration was focal, predominantly localized at the periphery of the fat graft in the tissue remodeling zone. The median count of lymphocytes and macrophages was 2.5 cells (IQR: 0–6.5). In the group with both preoperative and intraoperative PRP treatment, isolated lymphocytes and macrophages were noted, mainly in the area of contact with surrounding tissues. The median count of immune cells was 0 (IQR: 0–4.8).

#### 3.2.3 Comparative histomorphometric characteristics of angiogenesis

In the 1st and 2nd groups (without PRP treatment of the recipient site), microvessels were unevenly distributed, predominantly localized in the paracentral zones of the fat autograft. The median number of vessels in Group 1 was 2 (IQR: 1–2), and in Group 2, it was 2 (IQR: 1–3.25). In both cases, the vascular network had a focal pattern, with microvessels clustered in areas of tissue remodeling.

In the group with PRP treatment of both the graft and the implantation site, the microvascular network was more evenly organized, with vessels located in both the central and paracentral zones of the graft. The median number of vessels was 6 (IQR: 4–6).

## 4 Discussion

In this study, a comparative assessment of the survival of a fat autograft with the characteristics of macroscopic and histological patterns of the central and paracentral zones of the graft was carried out during preoperative PRP treatment of the implantation zone and intraoperative PRP treatment of the graft in an experiment on rabbits. The study demonstrated the effect of preoperative PRP treatment of the implantation area on several key parameters that determine the operation’s success.

First, we demonstrated that the use of preoperative PRP treatment at the implantation site improves the survival of the fat autograft compared to the group without PRP. (p = 0.0001 and p = 0.0001 for weight and volume, respectively) and the group with intraoperative PRP treatment of the fat graft and without preoperative PRP treatment of the implantation site (p = 0.015 and p = 0.01 for mass and volume, respectively).

Several animal studies have previously demonstrated a positive effect of PRP on fat graft outcome (Felthaus et al., 2017; Liao et al., 2014; Oh et al., 2011; Por et al., 2009; Rodríguez-Flores et al., 2011; Baykara et al., 2022). We believe that in our study, the mechanism of improving the survival of fat tissue may be associated with a mutual synergistic enhancement of the effect of PRP treatment of the implantation site preoperatively and fat graft treatment intraoperatively.

Secondly, we showed that preoperative PRP treatment of the implantation zone improves the histological pattern of the fat autograft due to less fibrosis and minimization of inflammatory activity (p < 0.01). In Group without PRP, the central and paracentral areas of the implant not occupied by intact adipocytes were occupied by fibrosis or cysts after 3 months. These vacuoles and cysts arising from destroyed Adipocytes can be resorbed by macrophages in the future and contribute to the development of fibrosis. Since we observed a significantly higher proportion of these unwanted tissues in fat grafts without preimplantation PRP preparation, we can assume a more significant decrease in the volume of fat grafts in Groups without PRP treatment of the implantation site over a longer follow-up period.

Histomorphometric analysis of the reparative pattern did not reveal an active inflammatory, allergic, or necrotic pattern in the implantation area using PRP treatment of the graft and the implantation site. In the Group with preoperative PRP treatment of the implantation site and PRP treatment of the fat graft, the number of immune-inflammatory cells was significantly lower compared to the Group without PRP (p = 0.015). The PRP-graft complex is biocompatible and does not induce an immunological response from a microorganism. The increased concentration of factors in PRP may play a crucial anti-inflammatory role by suppressing the pro-inflammatory factor, improving the fat graft’s survival. This is consistent with scientific evidence that PRP has anti-inflammatory properties that can reduce inflammation and swelling that contribute to fat graft degeneration (El-Sharkawy et al., 2007), and also exhibits immunomodulatory properties (Troha et al., 2023; Niemann et al., 2023; Osterman et al., 2015).

Thirdly, the results of this study convincingly confirm the authors’ hypothesis that the use of the preoperative PRP treatment method of the implantation zone increases the vascularization of the implantation zone. This, in turn, improves the survival rate of the fat graft. In the analysis of angiogenesis, we found that the relative number of microvessels in the Group with preoperative PRP treatment of the implantation site and PRP treatment of the fat graft was statistically significantly higher than that of the Group without PRP (p = 0.005) and the Group with only intraoperative PRP treatment of the fat graft (p = 0.01). In the group with preoperative PRP treatment of the implantation zone, microvessels had a relatively uniform distribution in the fat autograft’s central and paracentral zones. Moreover, active angiogenesis was uneven and chaotic in the comparison groups, mainly in the periphery. It is important to note that a volume of 3 mL in the subcutaneous space model of the rabbit ear allows for the simulation of volume-dependent ischemic stress, characteristic of large-volume fat grafting in challenging clinical conditions. The graft volume could contribute to the formation of hypoxic zones within the graft, which explains the observed differences in vascularization and resorption between the groups with and without preoperative PRP preparation of the implantation site.

The data obtained are consistent with the results of previous studies showing that PRP causes increased angiogenesis (DeRossi et al., 2009; Findikcioglu et al., 2012; Takikawa et al., 2011), increasing the density of blood vessels and improving their distribution (Li et al., 2020; Nishimura et al., 2000; Nakamura et al., 2010; Pires et al., 2010). The improvement in angiogenesis may be related to the growth factors present in PRP.

Tissue vascularization is one of the key conditions for preserving a fat graft. Immediately after grafting, the grafted fat tissue undergoes a state of acute ischemia. Ischemia is the leading cause of adipocyte death in fat grafts and can render cells nonviable within 24 h (Eto et al., 2012). Combining fat grafts with PRP increases fat grafts’ revascularization and the proportion of surviving cells. Initially, cells survive due to plasma diffusion of nutrients and oxygen from surrounding tissues. Neovascularization begins approximately 48 h after grafting due to ingrowth and reconnection of capillaries and vessels between the recipient’s bed and the graft (Liao et al., 2014).

The grafted part that is not vascularized during the first 3 days necrotizes and forms fatty cysts, fibrosis, and calcifications (Eto et al., 2012; Serra-Mestre et al., 2014; Kato et al., 2014) leading to a decrease in the volume of implanted fat. In our study, vascularization in the Group with PRP treatment of both the implantation site and the graft was significantly better compared to the Group with only intraoperative PRP treatment of the fat graft and without preoperative PRP treatment of the implantation site, as well as the group without PRP. This contributed to maintaining the volume and survival of the fat graft. This difference can be explained by the fact that preimplantation PRP treatment of the implantation zone, which causes preimplantation local action and activation of active factors (for example, cytokines growth factors), may be more effective for activating and accelerating neoangiogenesis in the first days after implantation of a fat graft, when the role and value of oxygen are most significant, which is crucial for the survival of fat tissue. The survival improvement mechanism of fat tissue may be associated with the mutual strengthening of two components, both preimplantation PRP preparation and intraoperative PRP treatment of fat graft, which mutually improve each other.

The results obtained in our study demonstrate that preoperative PRP treatment of the implantation site promotes better preservation of the fat graft through enhanced vascularization and reduced fibrosis. This effect is particularly important in large-volume fat grafting, where graft resorption can significantly impact aesthetic and functional outcomes. In other clinical scenarios, such as scar correction, the need for volume retention is less critical; however, the biological effect of the graft on surrounding tissues, which is improved by pre-implantation preparation of the recipient site, plays a key role. Thus, our results highlight not only the impact of PRP on graft volume but also its regenerative properties, which may be beneficial in various areas of plastic and reconstructive surgery.

The limitations of our study include the absence of a control model with saline injection, which could have demonstrated the baseline regenerative potential of the recipient site without PRP, as well as the necessity of blood collection from rabbits in the PRP group, which may have influenced ischemic preconditioning of the implantation site and the recovery process after surgery. Additionally, the assessment of fat graft survival was based solely on macroscopic and histological analysis. While these are valuable insights, it is important to acknowledge that other methods, such as IHC analysis, apoptosis detection, and proliferation assays, could have provided additional information for a more comprehensive evaluation of graft viability. These limitations should be considered when interpreting the results and planning future studies.

Thus, we have shown that preimplantation PRP treatment of the implantation zone improves the standard technique of intraoperative PRP treatment, increases the angiogenesis potential, and reduces the degree of hypoxic-ischemic, fibrotic and inflammatory damage in the fat autografting zone in an experiment on rabbits. The main effect of preserving the histological fat tissue pattern is associated with forming a loco-regional favorable microenvironment with active perfusion and diffusion potential of the stromal framework, which contributes to the improvement in the survival of the fat graft.

## Data Availability

The original contributions presented in the study are included in the article/Supplementary Material, further inquiries can be directed to the corresponding author.

## Abbreviations

PRP, platelet-rich plasma.
