# Supplementary figures and images for: PRP pre-treatment of the implantation zone improves the survival rate of fat autograft
Source: Front Bioeng Biotechnol. 2025 Apr 11;13:1545419. doi: 10.3389/fbioe.2025.1545419 (PMC12021894; doi:10.3389/fbioe.2025.1545419)

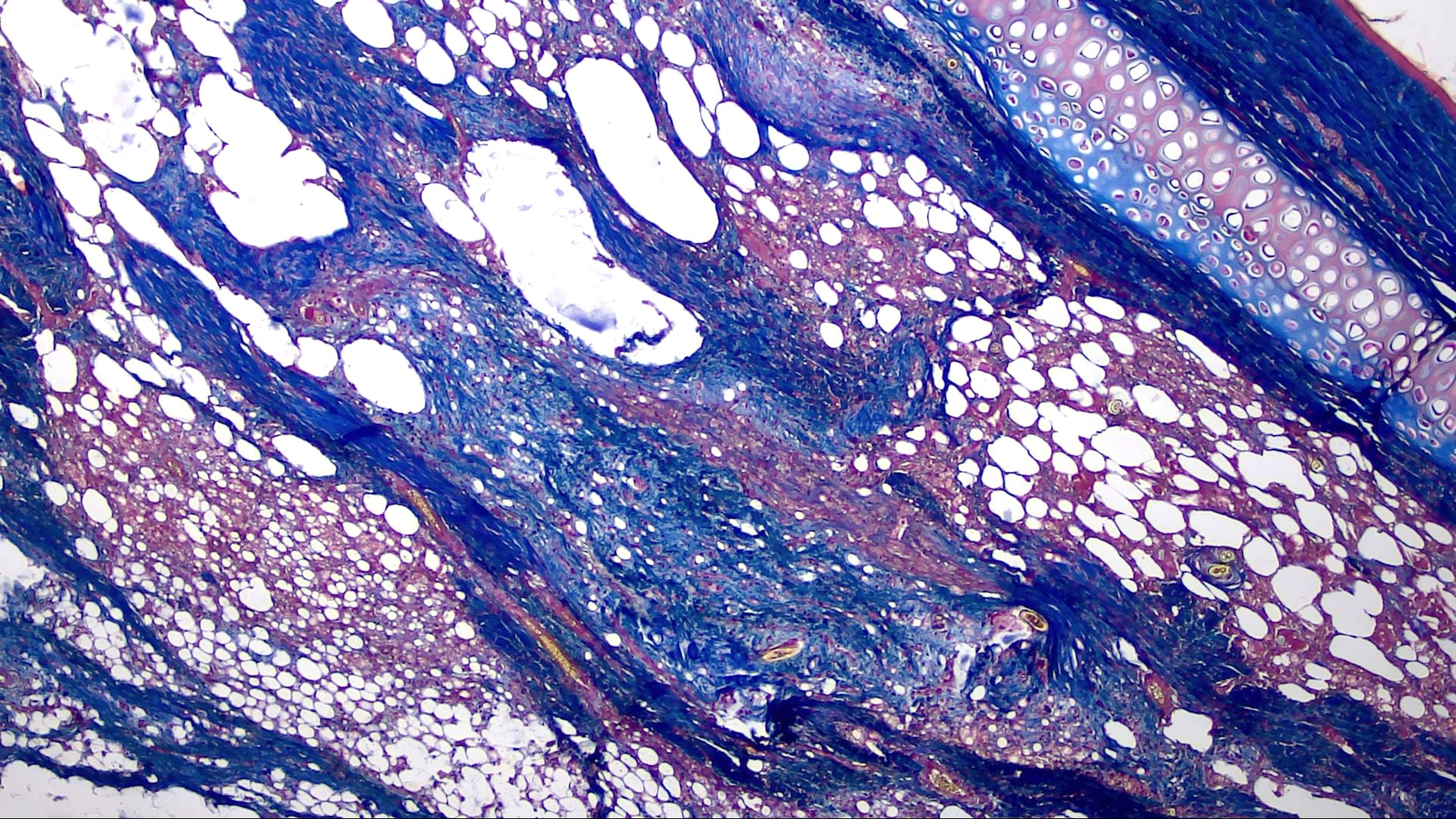

Supplement: Supplementary file 1 [file Image3.jpeg]

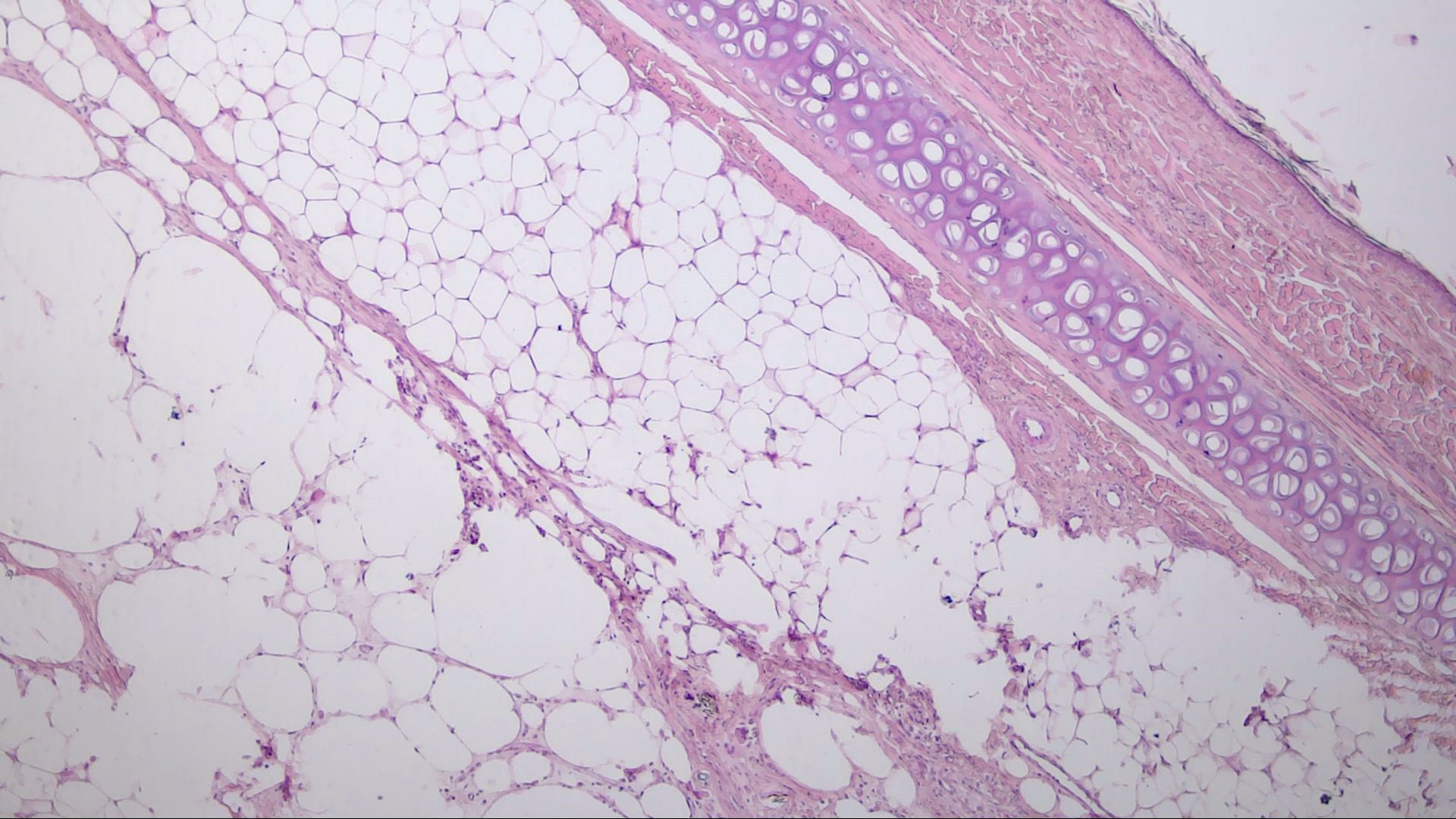

Supplement: Supplementary file 2 [file Image1.jpeg]

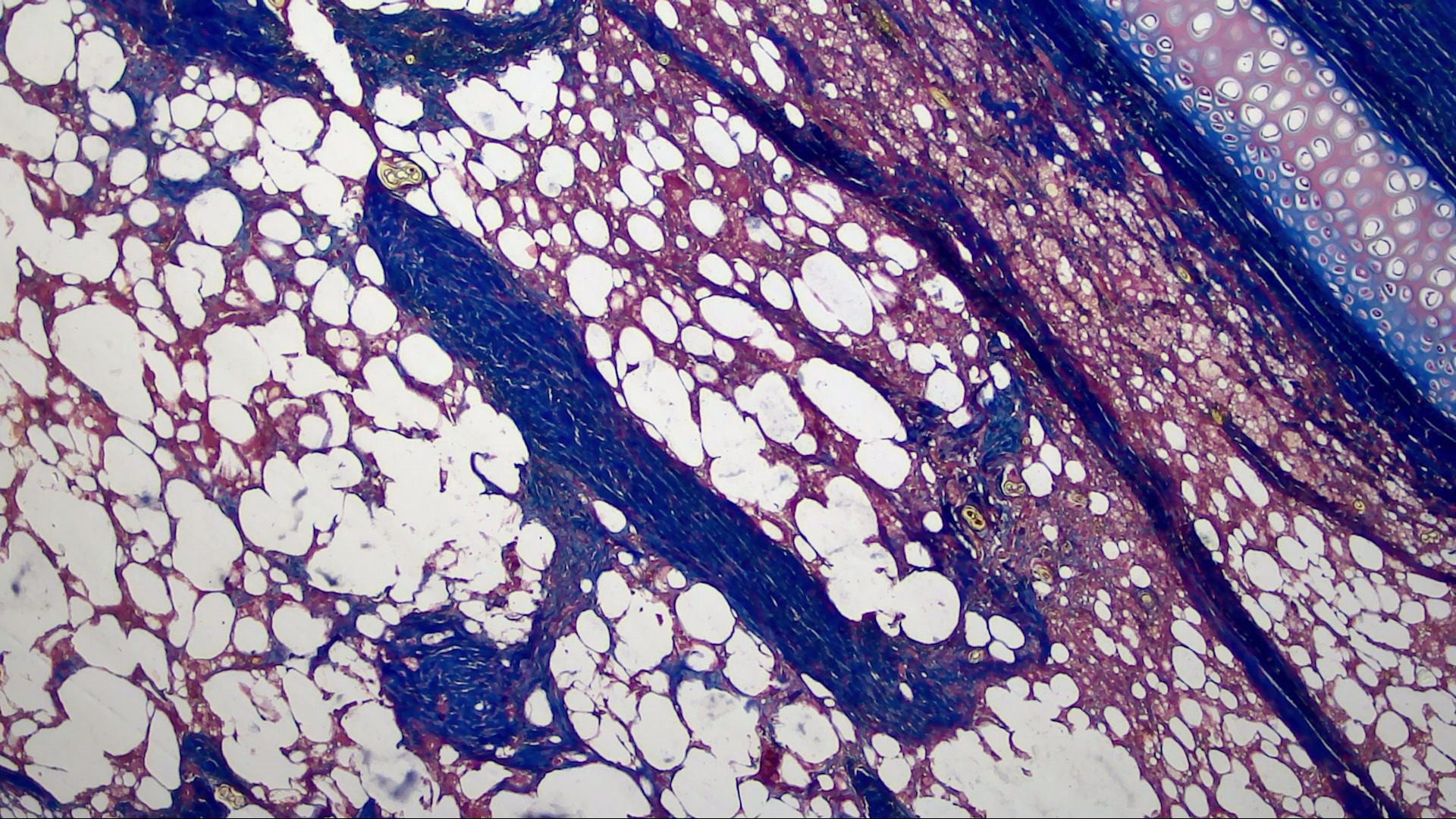

Supplement: Supplementary file 3 [file Image4.jpeg]

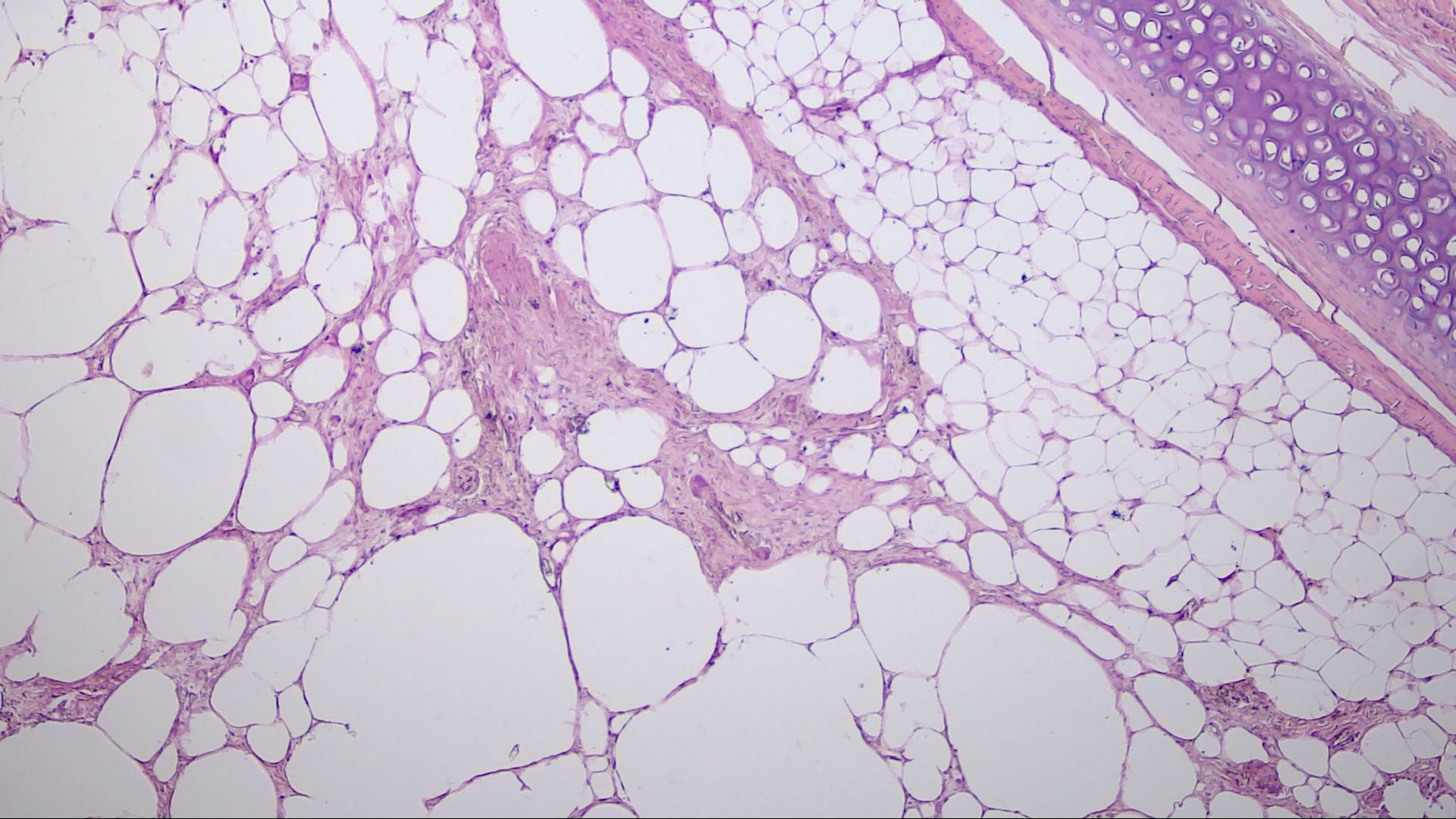

Supplement: Supplementary file 4 [file Image2.jpeg]

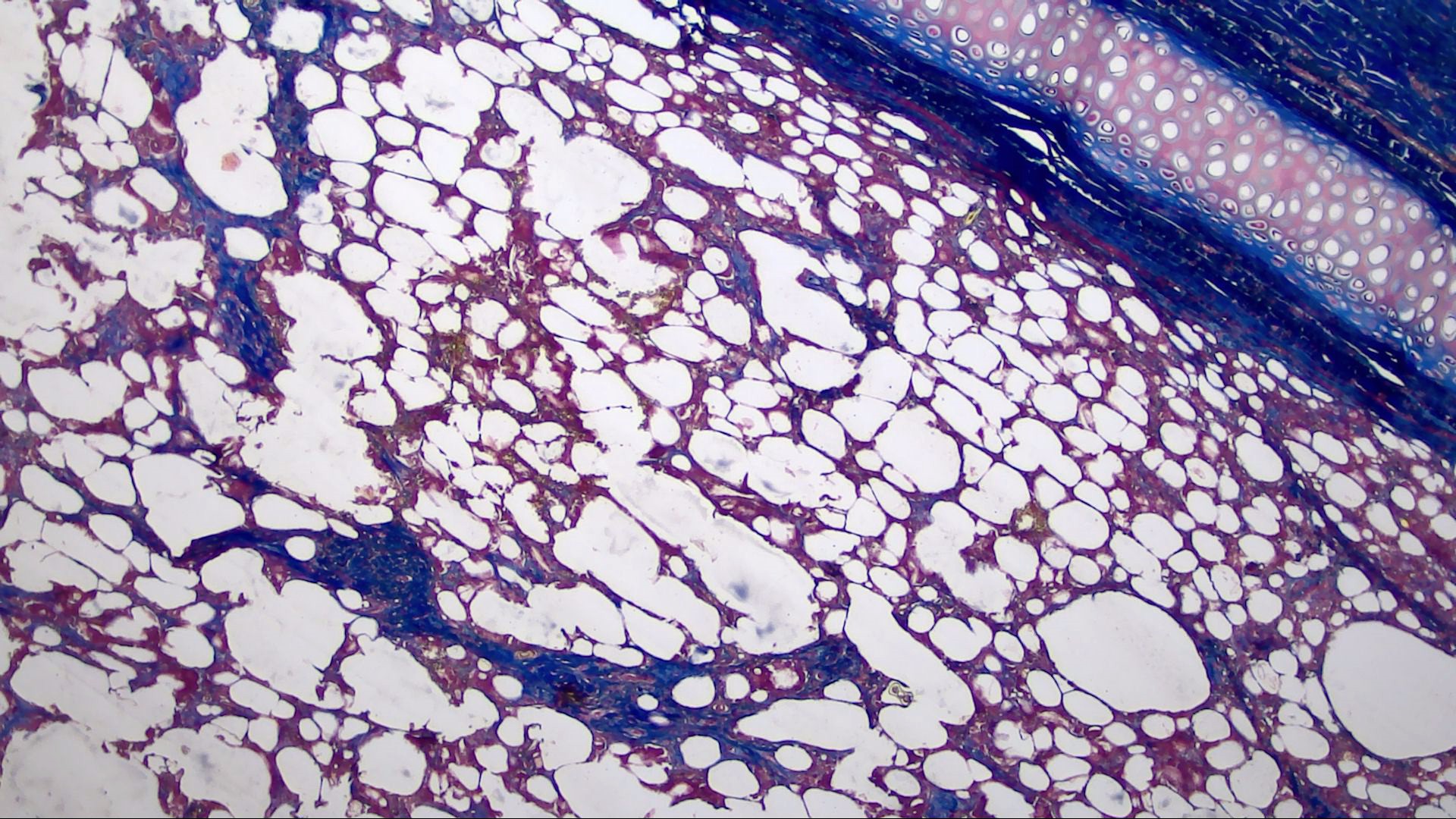

Supplement: Supplementary file 5 [file Image5.jpeg]

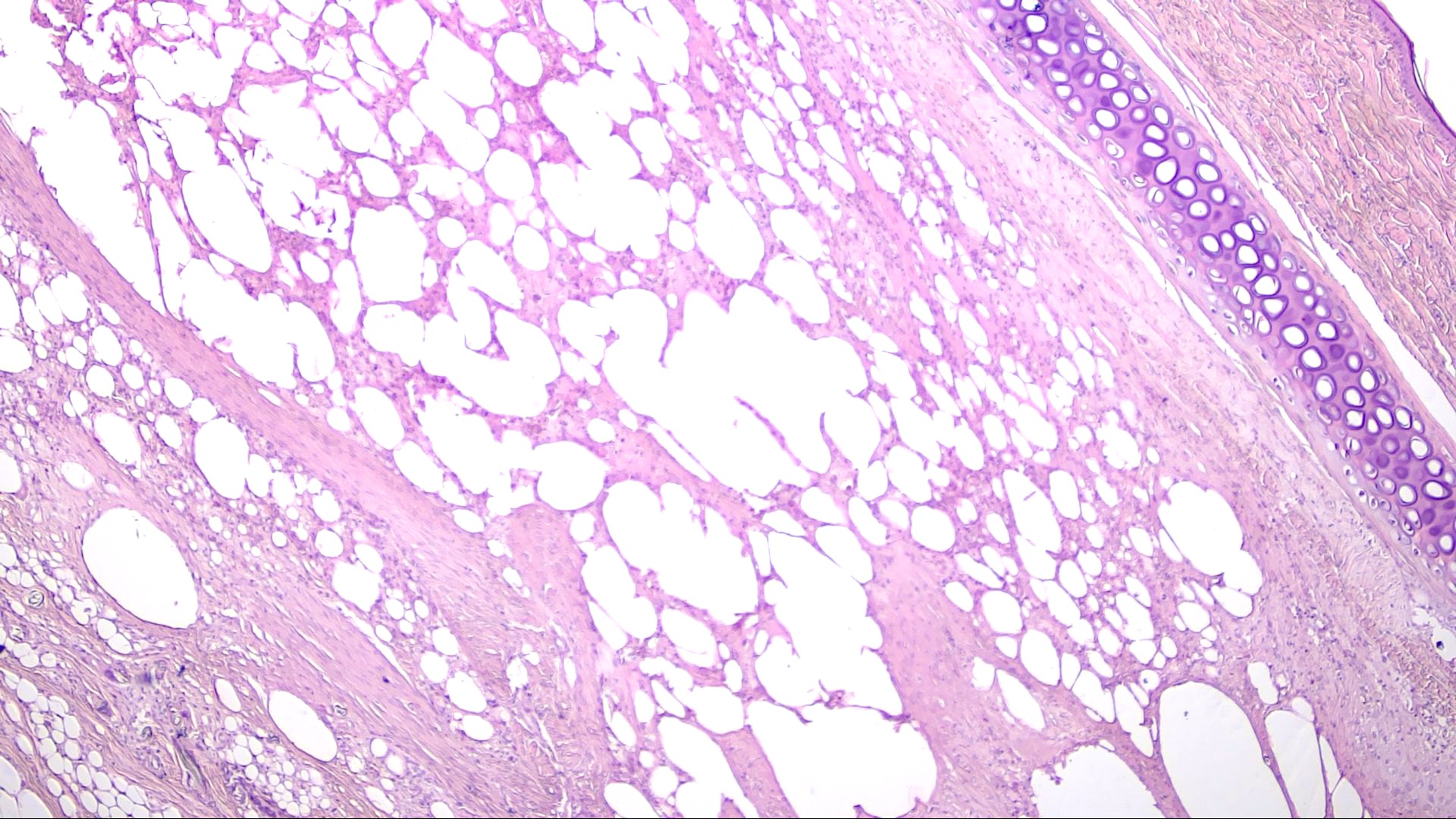

Supplement: Supplementary file 6 [file Image6.jpeg]
